# Utilization of Food Waste and By-Products in the Fabrication of Active and Intelligent Packaging for Seafood and Meat Products

**DOI:** 10.3390/foods12030456

**Published:** 2023-01-18

**Authors:** Maryam Adilah Zainal Arifin, Noranizan Mohd Adzahan, Nur Hanani Zainal Abedin, Małgorzata Lasik-Kurdyś

**Affiliations:** 1 Department of Food Technology, Faculty of Food Science and Technology, Universiti Putra Malaysia, Serdang 43400, Malaysia; 2 Department of Food Technology of Plant Origin, Faculty of Food Science and Nutrition, Poznan University of Life Sciences, 60-624 Poznan, Poland

**Keywords:** food waste, intelligent packaging, active packaging, industrial relevance, valorization

## Abstract

Research on the utilization of food waste and by-products, such as peels, pomace, and seeds has increased in recent years. The high number of valuable compounds, such as starch, protein, and bioactive materials in waste and by-products from food manufacturing industries creates opportunities for the food packaging industry. These opportunities include the development of biodegradable plastics, functional compounds, active and intelligent packaging materials. However, the practicality, adaptability and relevance of up-scaling this lab-based research into an industrial scale are yet to be thoroughly examined. Therefore, in this review, recent research on the development of active and intelligent packaging materials, their applications on seafood and meat products, consumer acceptance, and recommendations to improve commercialization of these products were critically overviewed. This work addresses the challenges and potential in commercializing food waste and by-products for the food packaging industry. This information could be used as a guide for research on reducing food loss and waste while satisfying industrial demands.

## 1. Introduction

The Food and Agriculture Organization of the United Nations (2019) reported that one-third of all food produced is lost or wasted every year, causing a global economic loss of about USD 7.5 trillion annually [1]. The agricultural production stage has been identified to incur the highest amount of food waste (413 MT), followed by the postharvest stage (293 MT) and the food processing (148 MT), distribution (161 MT), and consumption (280 MT) stages [2]. Realizing the severity of this situation, the European Union took proactive measures by implementing the global Sustainable Development Goal (SDG) with SDG 2 aiming to ‘end hunger, achieve food security and improve nutrition and promote sustainable agriculture’. Additionally, SDG 12.3 aims to ‘halve per capita food waste at the retail and consumer levels, and reduce food losses along production and supply chains (including post-harvest losses) by 2030′ [3].

Food waste and by-products (FWBP) have been a concern for decades, but the urgency to address this problem intensified only recently. Based on Google Trends, interest in food waste as a topic doubled since December 2017 (50) and reached its peak in April 2022 (100). A Scopus trend also showed that publications on food waste increased almost two-fold from 2016 (n = 2595) to 2021 (n = 6356). This rising interest in food waste justifies the relevance and importance of this review to discuss the utilization of food waste in depth.

Waste utilization can be divided into three categories: (1) Reuse (using it for the same purpose), (2) recycle (converting to similar or lower-value products), and (3) upcycle/valorize (converting to higher-value products) [4]. Generally, the utilization of FWBP falls under the valorization or upcycle category, as FWBP has little possibility of being reused (since it is perishable) or recycled (food by-products, such as peels, cannot be recycled or regenerated as peels again). In terms of commodities, the highest amount of food waste in Europe comes from agricultural produce (especially fruits and vegetables). In some cases, the waste discarded from these processing industries reaches more than 50% (Table 1). Furthermore, the huge amount of waste and by-products generated from the fruit and vegetable processing industry generally has a significantly higher amount of bioactive compounds, such as antioxidants, fiber, and antimicrobials than the product itself [5,6]. Therefore, this review primarily focuses on FWBP from agricultural produce.

**Figure 1:**
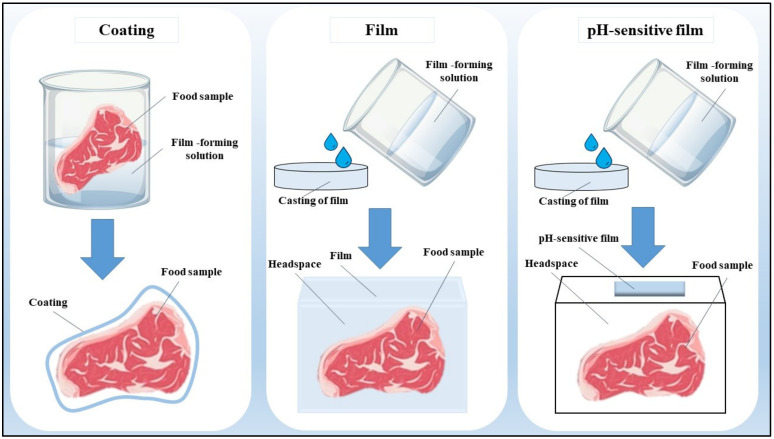
Applications of active packaging as coatings, film, and pH-sensitive film.

Regardless of the stage at which food is wasted, whether at agricultural production, postharvest, or during food processing, the massive number of valuable components in FWBP makes it a lucrative source for the development of other useful materials. One example is the development of active and intelligent packaging in the packaging industry. Active packaging incorporates active compounds, such as antioxidants and antimicrobials that interact with the packaged product or the headspace to maintain its quality or increase the shelf-life of products [16]. Active packaging protects food from spoilage by gradually releasing active compounds from the packaging to the food product, thus requiring a lower amount of active compounds compared to direct incorporation into food [17]. Normally, active packaging is applied in the form of a coating (dipping the product into a liquid film-forming solution), a film (a thin layer of solid laminate, such as plastic), [18,19] and a pH-sensitive film through the incorporation of an intelligent indicator [20] (Figure 1). These films and coatings could increase the shelf life of foods by exhibiting active properties (such as antioxidant and antimicrobial) and good barrier properties (such as water, light, and gas barriers) [21].

Recently, StixFresh created an antimicrobial sticker using a 100% natural substance rather than synthetic substances, and this has paved the way for natural products to be used as active packaging. This product shows that the concept of active packaging is widely accepted in the food industry, and there is potential for substituting synthetic active compounds with natural materials derived from FWBP. However, the relevance of using FWBP in the industry remains unanswered. Since this research has been carried out in the laboratory, the practicality, relevance, and functionality of FWBP in the packaging industry are still ambiguous, thus lowering its potential to be commercialized. Therefore, this review aims to explore the utilization of FWBP in the packaging industry and clarify the relevance of current research from both academic and industrial viewpoints. Specifically, this article discusses (1) the development of active and intelligent packaging using FWBP (antioxidant, antimicrobial, and pH-sensitive films); (2) the application of active and intelligent packaging to food products (seafood and meat); (3) consumer acceptance of meat and seafood products packaged in active and intelligent packaging; and (4) challenges and recommendations to improve the commercialization of FWBP in the food packaging industry. This review could provide insights for tailoring research toward satisfying industrial demand and emphasizing the potential and applicability of FWBP in the industrial sector.

## 2. Antioxidant Packaging

Antioxidants can delay the lipid peroxidation process by scavenging free radicals, which helps reduce food spoilage [22]. Antioxidant packaging is a type of active packaging developed by incorporating antioxidants into packaging materials. According to Almasi et al. [19], the active compound-releasing mechanism of active packaging can be divided into three types: (1) Diffusion-induced release, in which the active compounds are released from the polymer matrix into the food and are affected by the chemistry, porosity, and permeability of the film; (2) swelling-induced release, in which the active compound diffuses when its diffusion coefficient increases, as induced by the swelling of the polymer matrix; and (3) disintegration-induced release, in which the active compound is released when the polymer matrix is degraded, cleaved, or destroyed.

Although antioxidant packaging can be developed using synthetic antioxidants, such as butylated hydroxyanisole and butylated hydroxytoluene, FWBP utilization has emerged in antioxidant packaging. Recent studies have demonstrated the development of active packaging from FWBP, such as pomace, peels, and seeds (Table 2). Examples of studies that used plant pomace are apple pomace (containing phenolic acids and flavonoids) [23] and blueberry and red grape skin pomace (containing flavonoids and phenols) [24]. Plant peels (or shells, skin, and hulls), such as grapefruit peels (containing coumarin and flavonoids) [25], peanut shells and skin (rich in polyphenols, flavonoids, and amino acids, such as 5,7-dihydroxychromone, eriodictyol, 3′,4′,7-trihydroxyflavanone, and luteol) [26], prickly pear peel (containing betalain), plant seeds, such as grapefruit seeds (containing phenolic compounds), and black soybean seed coats (abundant in anthocyanins) [27] have been used for the development of antioxidant packaging.

Conventional biodegradable packaging materials, such as polysaccharides (cellulose and starch) and proteins (gelatin, soy protein isolate, and whey protein) are normally used as packaging materials for antioxidant films [46]. However, recently, studies have explored the development of antioxidant films entirely from a single source. Melo et al. [40] utilized compounds derived only from mango kernels. In this research, mango kernel starch (used as a biopolymer material), mango kernel extract (a source of antioxidants and an ultraviolet absorber), and mango kernel fat (a source of hydrophobic ingredients) were developed. Mango kernel starch is a novel source of biopolymer material, and it has a comparable amylose content to corn starch. The linear structure of amylose is known to contribute to the development of a rigid film as it can form hydrogen bonds more readily than branched amylopectin [40,47]. The 2,2-diphenyl-1-picrylhydrazyl inhibition of this type of film (>90%) is the highest compared to other films using mango waste and by-products, such as the peel (84.50%) [38] and mango kernel extract (64%) [48]. These differences are probably due to the type of biopolymer used (generally other types of biopolymers exhibit negligible to minimum antioxidant properties, in contrast to mango kernel starch) and the variety of mango that was used.

FWBP are also hugely contributed by food-related industries. To reduce the problem, the development of active packaging from a single source provides a sustainable opportunity to fully utilize FWBP. For example, in the fruit juice industry, producers of products, such as mango juice could develop active packaging entirely from their own industrial waste and by-products without the need to purchase other materials (biopolymers and active compounds) from other sources. Unconventional sources, such as mango kernel could be explored to produce better biopolymer materials that also have antioxidant properties. However, the film formulation must be tailored to specific applications, as the tensile strength, water vapor permeability and active functions of films can vary, depending on the percentage of biopolymer material and active compounds included.

Oliveira et al. [49] created an antioxidant film made entirely from potato and coffee processing waste and by-products. Potato starch film (recovered from broken potato slices) incorporated with 1%, 5%, and 10% coffee silverskin (a by-product of the coffee-roasting industry) showed good optical, mechanical, and barrier properties. Additionally, coffee silverskin extract tripled the antioxidant activity of the film as compared to a control experiment. Similarly, de Moraes Crizel et al. [50] developed a novel active biodegradable film from food-grade industrial waste. The packaging material was made from discarded gelatin capsules from nutraceutical capsule production, while the active compound and fiber were extracted from blueberry waste. Seven formulations were used in this research: Three formulations of blueberry pomace dietary fiber (0.05, 0.10, and 0.15 g/mL), three formulations of blueberry pomace extract (30, 40, and 50 mL), and control film without the addition of fiber or extract. The antioxidant activity of the film was observed to be stable for up to 21 days, and it exhibited excellent protection against lipid oxidation in sunflower oil for 13 days. Since lipid oxidation is a major problem in the food industry due to its detrimental effects (organoleptic changes, nutritional loss, and formation of toxic compounds), the antioxidant effect of this film could provide a major benefit to the industry.

The functionality of antioxidant packaging and its releasing mechanism is largely dependent on the incorporated active compound and its interaction with the biopolymer matrix. For example, Kurek et al. [24] demonstrated the different antioxidant activities of blueberry pomace extract and red grape skin pomace extract in chitosan (CH) and carboxymethyl cellulose (CMC) films. Interestingly, the blueberry pomace extract/CH film showed a higher total phenolic content (up to 50% higher) than red grape skin pomace extract/CH, yet the opposite result was shown in the CMC. This variation is primarily due to the bonding of phenols in different types of biopolymer matrices. This is in agreement with the antioxidant activity of mango kernel extract in two types of protein-based films, soy protein isolate and fish gelatin films [51]. The differences in the structure of biopolymer materials affect the bonding of active compounds and the biopolymer matrix. The globular structure of soy protein isolate films allows for less protein-phenolic interaction to occur; thus, a higher amount of free phenolic compound is available to react in the antioxidant analysis compared to the linear structure of fish gelatin films. However, according to Kuai et al. [52], factors including the free volume and mobility of the polymer chain segments can control the release rate of antioxidants in different types of polymers. Antioxidants migrate slower in less porous and low free-volume matrix due to more tortuous diffusion pathways. Therefore, these factors should be adjusted to the desired release rate by selecting the appropriate active compound and biopolymer matrix for the development of active and intelligent packaging.

## 3. Antimicrobial Packaging

Antimicrobial packaging has been developed to alleviate the problem of microbial spoilage in food [53]. This type of packaging can be classified depending on its development method into several categories: (1) Antimicrobial compounds in sachets; (2) antimicrobial compounds in a polymer matrix; (3) coating on the surface of polymers; (4) inherent antimicrobial polymers; and (5) entrapping antimicrobial compounds in polymers by ion or covalent linkages [54,55]. Antimicrobial compounds, such as polyphenols are known to kill microorganisms by destroying the cell wall, causing the cytoplasmic contents to leak out, and disrupting dehydrogenases in the mitochondria and plasma [56].

Research on antimicrobial packaging and FWBP (Table 2) has focused on the use of pomace, such as grape pomace (containing polyphenols) [57], plant peels (or shells, skin, and rinds), such as pomegranate rind (rich in tannins and polyphenols) [58], apple peel (containing phenolic acids, flavonols, flavon-3-ols, anthocyanins, and dihydrochalcones) [59], as well as plant seeds, such as date palm seed (rich in polyphenols) [60] and grapefruit seeds (consisting of polyphenols, flavonoids, citric acid, ascorbic acid, tocopherol, and limonoid) [61].

CH is the preferable biopolymer material for antimicrobial packaging as it exhibits antimicrobial function [39]. Published literature shows that the type of carrier is predominantly in the form of a film rather than a coating or encapsulation. Antimicrobial films based on CH and FWBP have successfully been developed using agro-industrial extract [62] and apricot kernel essential oil [63]. Furthermore, Poverenov et al. [64] developed a novel coating material (CH) from the valorization of mushroom waste (mushroom caps and champignon stipes) that exhibit antifungal activity. The extracted CH can be used as an alternative to animal-based CH, benefitting people with allergies as well as those with vegan and vegetarian preferences. Based on the permissible level of total aerobic microbial count (7 log CFU/g) and mold and yeast (2.7 log CFU/g) in ready-to-eat produce, this coating was able to prolong the shelf-life of fresh-cut melon to up to 11 days. In the industry, the ability of the packaging to extend the shelf-life of a product could decrease economic loss; therefore, this type of coating could be beneficial in the food sector.

Biodegradable polymers are typically used for the film matrix that carries antimicrobial compounds. However, generally, biopolymers have poor mechanical and water barrier properties. Alternatively, Spiridon et al. [65] developed a biocomposite film using a commercially available biodegradable plastic, Ecoflex^®^, waste biomass, and starch to ameliorate this problem. The main highlight of this research was that commercialized biodegradable plastic could benefit from the addition of FWBP. The addition of biomass waste, such as celery fibers, poplar seed hair fibers, grape pomace, *Asclepias syriaca* fibers, and lignin was able to increase the tensile strength of the film up to 127.7%. In terms of antimicrobial potential, the addition of lignin and biomass waste imparted good antimicrobial effects against *Staphylococcus aureus* (*S*. *aureus*) and *Escherichia coli* (*E. coli*). In particular, grape pomace had the highest inhibition of *E. coli* due to the high content of aromatic structures. The results showed that the antimicrobial effect was more prevalent against Gram-negative bacteria (*E. coli*) than Gram-positive (*S*. *aureus*) due to the thicker peptidoglycans in Gram-positive bacteria that act as a barrier to the effective transmembrane transport of bioactive substances. The thinner peptidoglycan layer in Gram-negative bacteria imparts less resistance to hydrophobic compounds [66].

## 4. pH-Sensitive Film

Intelligent packaging, such as those containing pH indicators, gas indicators, and time-temperature indicators provides information to the consumer about the packaged product (safety, spoilage, or temperature change) [20]. However, in contrast to other complex and generally more expensive intelligent packaging, colorimetric pH indicator film has gained interest due to its simplicity, which is consumer-friendly and provides accurate real-time information, and is a non-destructive and non-invasive [67,68] pH-sensitive film that involves a color alteration as a response to pH changes in the packaged food [69]. A pH change, especially in proteinaceous foods, is normally triggered due to food spoilage (microbial growth or oxidation); therefore, pH-based films are deemed suitable for use as a universal indicator of the condition of food [70]. Colorimetric pH-sensitive film is included in this review paper as it is directly linked to the functionality of antioxidant and antimicrobial films. In the previous section, the functionality of antioxidants and antimicrobial film could only be demonstrated through in vitro analyses, but its development into pH-sensitive films could show a visible effect through a colorimetric response.

Traditional pH indicator film is developed by embedding synthetic indicator dyes that are sensitive to a certain range of pH, such as bromocresol purple (pH 5.2 to 6.8), methyl red (pH 4.4 to 6.2), and bromocresol green (pH 3.8 to 5.4) in packaging materials [68,71]. However, the toxicity of synthetic dye has prompted numerous studies to extract color compounds from FWBP as a safer alternative (Table 3). Several types of pH-sensitive color compounds from FWBP have been used in pH-sensitive films, such as curcumin (from turmeric residue) and betalain (from dragon fruit peel), but the majority of literature has focused on anthocyanin, probably due to its wide availability [72].

Anthocyanin is described as a water-soluble pigment that is natural, non-toxic, and a good indicator of pH changes [78]. Gutiérrez et al. [79] developed a pH-sensitive film using unconventional guinea arrowroot starch incorporated with grape waste (in flour and extract form) from the waste of the wine-making industry as the source of anthocyanin. Interestingly, only the starch/grape flour film was able to show a pH-sensitive function, while starch/grape extract did not. Furthermore, starch/grape flour can be used for low acidity samples (pH 1) to neutral samples (pH 7), but it disintegrated when exposed to high alkaline samples (pH 13) due to the reduction in hydrogen bonding between starch and glycerol, resulting in swelling and starch gelatinization. This is in contrast to the majority of pH-sensitive films, which were successfully developed using extracts, such as those from blueberry and blackberry [80] as well as raspberry pomace extract (RPE) [74]. The film containing blueberry extract showed color changes from bright red (at pH 2 to 4) to blue/green (at pH 5 to 7) and dark green (pH 10 to 12), while the color of films with blackberry extract was determined to vary from bright red (pH 2 to 4) to violet (pH 5 to 7) and dark blue (pH 10 to 12). Similarly, film containing RPE changed colors from pink-red-brown-blue-dark green (pH 1 to 13). Differing results are due to the type of film development methods. Starch/grape waste (flour and extract) films were developed using a harsher thermo-molding method, while films containing blueberry, blackberry, and RPE were fabricated using the solvent casting method, which is a significantly milder method on the active compound and biopolymer matrix. The pigment in extract is significantly more readily available (as compared to the flour form), and this exposes it to destruction by the harsh processing conditions of thermo-molding. However, this did not apply to grape flour, as the flour matrix protects the pigments during processing. This phenomenon was not significant for the solvent casting method, as processing did not include a high shear force and pressure. Therefore, the development method is a crucial factor to consider for pH-sensitive film fabrication to ensure its functionality.

While previous research used pomace, peel, and seeds from food waste, recent research by Eze et al. [70] utilized an unconventional pH indicator, which is, broken riceberry. Riceberry is a unique type of rice produced in Thailand, and processing generates 1200–1800 tons of broken rice per harvest season. In that research, a holistic approach to develop a pH-sensitive film was used, as it considered all aspects of the film, including color changes, and mechanical, water barrier, and antioxidant properties. This study also focused on the in vitro cytotoxicity of the film, which is an uncommon analysis in other publications in this field. The anthocyanin and hydrophobic components of riceberry impart a good colorimetric response and physical properties to the film. The film showed an intense color change from pH 2 to 12 (orange-red to yellow), had an eight-fold higher antioxidant ability compared to the control, had high hydrophobicity, and improved mechanical properties (tensile strength of 23.33–34 MPa). Most importantly, the film was also nontoxic, cytocompatible, and safe for food packaging. This highlights the potential of exploring novel sources of pH indicators and the type of analysis that could be incorporated, such as cytotoxicity tests, to enhance research on FWBP in packaging.

## 5. Application of Packaging Incorporated with FWBP on Food Products

The application of active packaging on food products is important to determine the efficiency and functionality of active packaging. However, it is crucial to ensure that a suitable type of food is used based on the function of the active packaging. For example, antimicrobial packaging is used in foods with a high rate of microbial spoilage and low-shelf-life products [81], such as seafood, meat, confectionery, and fresh-cut fruit. pH-sensitive film is suitable for application on food that shows pH changes due to microbial spoilage or oxidation [82]. Alternatively, antioxidant packaging can have a smaller scope for usage, since it is designed mainly for inhibiting lipid and protein oxidation in high-fat and protein-containing food. The following sections will focus on seafood and meat products as they are suitable for packaging with antimicrobial, pH-sensitive film, and antioxidant packaging.

### 5.1. Seafood

Proteolysis of the flesh produces volatile amines, such as ammonia, di- and trimethylamine (collectively known as total volatile basic nitrogen—TVBN), which could be used to indicate the freshness of seafood and meat. On the other hand, the peroxide value (PV) and thiobarbituric acid reactive substances (TBARS) are indicators of primary and secondary oxidation, respectively [83]. These three analyses have been widely used to indicate the quality of seafood and meat during storage.

Although the literature in this review paper is divided into different types of packaging, such as antimicrobial, antioxidant, and pH-sensitive films, packaging could have a combination of these effects on food products. For example, Wu et al. [75] developed an intelligent film based on CH/oxidized chitin nanocrystals containing black rice bran that showed both antioxidant activity and pH-sensitive capability. Two types of film, namely, COB-3 (containing 3% extract) and COB-5 (containing 5% extract), were used to detect the spoilage of pomfret and shrimp for 24 h. COB-3 changed from purple to grey, while COB-5 changed to brown at the end of storage. These films also decreased the TVBN of seafood due to their antioxidant content, which prevents the production of volatile nitrogen-containing compounds. Similarly, Ge et al. [20] fabricated an intelligent packaging from black rice bran and gelatin/oxidized chitin nanocrystals to monitor the freshness of shrimp and hairtail for 24 h. Films with a lower content of extract changed from purple to grey, while those containing a higher content changed to brown, similar to the findings of Wu et al. [75]. The antioxidant effect of these films also helps reduce levels of TVBN by decreasing the formation of alkaline substances. Ardiyansyah et al. [84] developed a pH-sensitive film by incorporating betacyanin into a glucomannan-polyvinyl alcohol matrix. The result showed a color change from purple (on the 1st day) to yellow (on the 8th day) of fish storage. This color change was aligned with the increase in TVBN on the 8th day, which exceeded the spoilage limit (39.74 mg/100 g). These studies illuminate the ability of colorimetric pH-sensitive film in monitoring the condition of packaged products and at the same time increasing the shelf-life of food, which will be beneficial in the industry.

Shruthy et al. [85] isolated cellulose nanoparticles from potato peels to reinforce a polyvinyl alcohol-based film for shrimp preservation. The film combined with fennel seed oil was able to maintain the quality of unpeeled and minimally processed prawns for up to 63 days while frozen. Similarly, Mohebi et al. [86] have examined the effect of pomegranate peel extract (POP) combined with *Ziziphora clinopodioides* essential oil (ZEO) and cellulose nanoparticles (CN) in two types of packaging materials, CH and gelatin films. Shrimp packed in CH/1% ZEO/1% POP/1% CN showed superior organoleptic scores after 11 days and higher antibacterial effectiveness against psychrotrophic bacteria, *Pseudomonas* spp., *Pseudomonas fluorescens*, *Shewanella putrefaciens*, Enterobacteriaceae, lactic acid bacteria, *Listeria monocytogenes*, and total viable count. This shows that FWBP incorporation into different types of biopolymers successfully prolongs the shelf-life of food and improves its organoleptic properties.

Rezaei et al. [87] compared the efficiency of the direct addition, edible coating, and biodegradable film of ZEO, apple peel extract (APE), and zinc oxide nanoparticles (ZnO) in extending the shelf-life of sauced silver carp fillet. All treated samples had a decreased TVBN value, trimethylamine nitrogen, pH, and PV. ZEO/APE/ZnO treatments showed antibacterial effects on total viable count, psychrotrophic bacteria, *Pseudomonas* spp. *P. fluorescens* count, H_2_S-producing bacteria, and Enterobacteriaceae. The edible coating also had appropriate sensory properties (odor, color, and overall acceptability). The result showed that the antibacterial effect is strongest in the following sequence: Edible coating > direct addition > biodegradable film. This result can be reviewed critically from two different perspectives: (1) Comparison of direct addition and active packaging (edible coating and biodegradable film) and (2) comparison between the edible coating and biodegradable film only.

**Figure 2:**
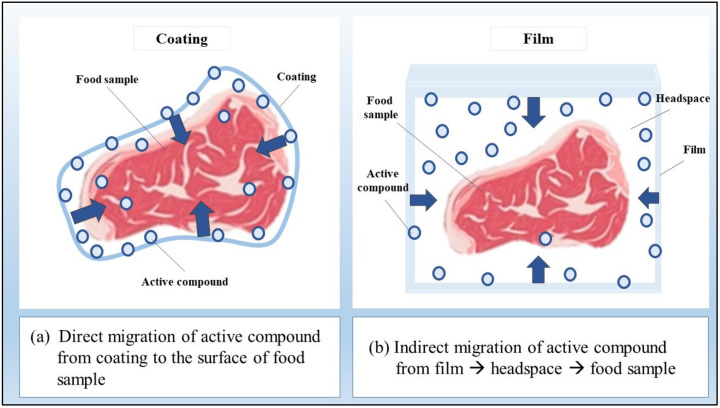
The mechanism of active compound migration from (**a**) coating and (**b**) film.

Theoretically, direct addition of active compounds, such as antimicrobial or antioxidant compounds are expected to show the highest inhibitory effects on food product (direct addition > edible coating > biodegradable film). This is due to the higher percentage of available active compounds that can react with the food product in the direct addition method compared to an edible coating or film (since active compounds in edible coating and films are entrapped in a polymer matrix; thus, there is a lower amount of free active compound) [88]. However, in this research, the higher antimicrobial effect of edible coating compared to direct addition method is probably due to the fact that edible coating provides gradual release of active compounds to the food product. For example, the experiment was conducted over 2 weeks; thus, it is possible that the active compounds in the direct addition method were used shortly after application. On the other hand, the controlled release of active compounds from edible coating helps it to exhibit an antimicrobial effect for a longer time. Comparing edible coatings and biodegradable film, the better antibacterial effect for edible coatings is attributed to the direct contact of the edible coating with the food. On the other hand, biodegradable films rely more on the headspace between the food and the film (film-headspace-food indirect contact system), as shown in Figure 2 [52]. This is also related to the amount of volatile compounds that penetrate from the biodegradable film into the headspace to react with the food [52].

However, it is also important to note that the application of coating and film is related to the type of food. Generally, films are mainly used rather than coating, probably due to their suitability for a wider variety of products that are normally wrapped [89], such as confectionery, pastries, fruits, meat, seafood, and food that is normally purchased in bulk (such as sliced bread, grapes, and berries). However, a coating is deemed not suitable for food with a higher affinity for water, such as confectionery and pastries and food purchased in bulk (it is harder to coat an entire bunch of grapes rather than wrapping it in film). Therefore, the type of active packaging used in the food industry varies, depending on the nature of the food product.

### 5.2. Meat

Chicken is widely consumed around the world, but its high protein and lipid content exposes it to the oxidation process. Han et al. [32] developed a novel active packaging film from watermelon rind pectin and kiwifruit peel extract to be used as chicken thigh packaging. The developed film successfully lowered the PV (4.49 meq peroxides/kg) compared to the control (5.02 meq peroxides/kg). The active film also showed a 36% lower TBARS value compared to the control on the 9th day of storage. Another study by Sogut et al. [90] created a CH and polycaprolactone-based (PCL) monolayer and bilayer films containing grape seed extract (GSE) and nanocellulose (NC) for chicken breast fillet preservation. Samples wrapped in CH/PCL/GSE/NC bilayer films exhibited a 39% lower TBARS value than controls. This inhibition of lipid oxidation was mainly contributed by the GSE, which contains phenolic constituents. The combination of CH, GSE, and NC was also effective in decreasing the total mesophilic aerobic bacteria and total coliform bacteria growth. The control sample exceeded the 7 log CFU/g (accepted limit) after the 6th day of storage, whereas the samples packaged in CH/GSE/2% nanocellulose only recorded 5.30 log CFU/g after 15 days. Consequently, the bilayer films extended the shelf-life of chicken thighs for 3 to 6 days.

Beef is another important commodity that undergoes lipid oxidation and microbial spoilage. Priyadarshi et al. [91] developed a CMC-based film containing ZnO and GSE that exhibited both antimicrobial and antioxidant effects on beef. According to the International Commission on Microbiological Specifications for Foods, 7 log CFU/g is the microbial limit for meat products. In this study, the control sample exceeded this limit after the 15th day of storage, while beef packed in active film remained safe (5.9 log CFU) even after 15 days. The PV of beef wrapped in active film was also 88% lower than the control. This shows the high efficiency of the film in terms of inhibiting lipid oxidation and extending the shelf-life of beef. Wang et al. [45] fabricated a CH film containing apricot kernel essential oil (AKEO), which could preserve the quality of spiced beef. Spiced beef packed in film with 1% AKEO had a low PV (3.6 meq peroxide/kg) and TBARS (0.4 mg MDA/kg) compared to the control (PV value = 8.2 meq peroxide/kg and TBARS = 0.8 mg MDA/kg) on the 24th day of storage. The total carbonyl (TC) content of packed spiced beef was also 67% lower than the control at the end of storage. Overall, the film decreased the PV, TBA, and TC of spiced beef and improved the sensory properties of the beef. 

Yang et al. [74] developed a pectin/sodium alginate/xanthan gum composite film containing RPE that showed pH sensitivity. The color of the RPE in buffer solution changed from red (pH 1–3) to pink (pH 4–6), blue-purple (pH 7–10), and green (pH 11–13). The application of the film to pork skin showed a color change to blue after 6 h (TVBN = 9.62 mg/100 g) and brown after 12 h (TVBN = 15.55 mg/100 g). Since the TVBN value exceeded the spoilage limit (15 mg/100 g) of pork according to the Chinese Standard (GB 5009.288–2016), this film successfully indicated a colorimetric response to spoilage at 12 h. Similarly, Chi et al. [68] developed a pH-sensitive film from k-carrageenan and grape skin powder to indicate pork freshness. Interestingly, the grape skin was added directly without going through an extraction process, thus providing a simpler and cleaner process for the development of an active film. The color of the active film changed from purple to green, corresponding to an increase in the TVBN value of pork (from 8.23 to 14.63 mg/100 g) during 15 days of storage.

In summary, this section highlights the ability of FWBP incorporation into packaging to decrease lipid oxidation and microbial growth and improve the sensory properties of meat. pH-sensitive films show accurate colorimetric responses according to the spoilage rate. These studies could be the fundamental basis for upgrading the usage of FWBP for commercialization in the food packaging industry.

## 6. Consumer Acceptance of Meat and Seafood Products Packaged in Active and Intelligent Packaging

Numerous research studies have proven the ability of active and intelligent packaging in prolonging the shelf life and quality of packaged meat and seafood products. However, the usage of active and intelligent packaging could also negatively affect the sensorial properties, such as taste, texture, and color of the packaged food through the migration of active compounds from the packaging to the food. Therefore, it is important to ensure that sensory and organoleptic properties of foods packaged in active and intelligent packaging are acceptable to the consumers.

The sensorial and acceptability of ground beef patties packaged in ginger starch-based edible films incorporated with coconut shell liquid smoke (film + CSLS) were evaluated after 2 days of storage [92]. Eight semi-trained panelists in meat product evaluation rated the color, odor, flavor, and general acceptability of the cooked beef patties using a nine-point hedonic scale. The results showed that no significant differences were found in the general acceptability between the control and the samples. Ground beef patties packaged in films containing CSLS at 5%, 10%, and 15% showed overall acceptability scores of 7.14, 7.00, and 6.86, respectively, which passed the minimum overall acceptability limit (5). Therefore, the usage of film + CSLS has no negative effect on the sensory qualities of ground beef patties and is widely accepted by the consumer.

Similarly, Shin et al. [93] also studied the organoleptic properties of both raw and cooked beef patties but with a larger number of panelists (40). The sensorial properties of cooked beef patties that were coated with apple peel-based edible coating showed higher scores than the uncoated control samples. The active coating also improved the texture, taste, and overall acceptability of the cooked beef patties probably due to the presence of proteases in the coating that tenderized the meat. This contributes to the higher acceptability of coated meat as compared to control.

Ucak et al. [94] studied the sensorial effects of gelatin films (GF) enriched with 2% of citrus seeds extracts (CSE) on the quality of sea bass fillets during refrigerated storage for 15 days. Ten experienced panelists in fish evaluated the odor, texture, color, appearance, and overall acceptability using the nine-point hedonic scale. The result showed that fish samples coated with GF + CSE have higher sensory values as compared to the samples uncoated or coated with GF only. CSE imparted positive effects on the appearance, odor, color, and texture properties of the fish and was preferred by the panelists. According to sensory assessment, the organoleptic quality of fish can be improved, and the shelf life can be prolonged by 9 days as compared to the control.

Similarly, the organoleptic properties of minced trout fillet fish samples packaged in chitosan-gelatin film incorporated with ethanolic RGS and ZEO over a period of 11 days were evaluated [44]. Nine trained panelists evaluated the odor, color, and overall acceptability using a scale ranging from 1 (dislike extremely) to 10 (like extremely). Fish samples packaged in film containing ZEO, 1% and 2% RGS showed the highest scores up to the 11th day of storage. On the other hand, the untreated sample (control) had lower overall acceptability throughout the storage days due to the higher microbial growth as compared to fish samples packaged in the active packaging.

## 7. Challenges and Recommendations to Improve the Commercialization of FWBP in the Packaging Industry

**Figure 3:**
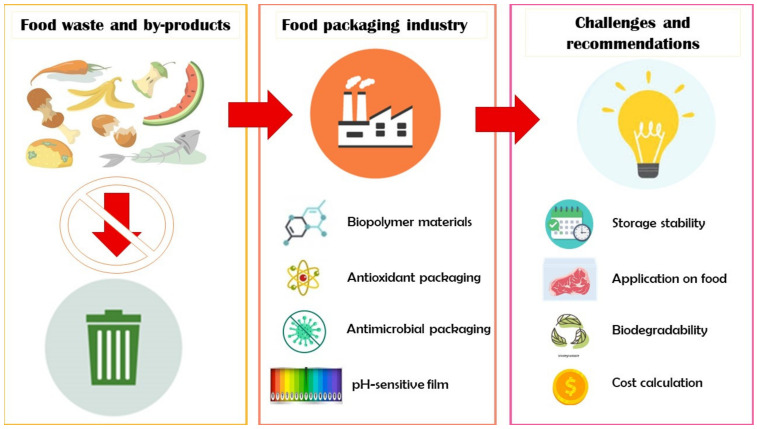
Application, challenges, and recommendations of food waste and by-products utilization for the food packaging industry.

The utilization of FWBP for the packaging industry has been successfully demonstrated in numerous studies by academia. However, challenges remain in terms of functionality, food application, biodegradability, and costs in the process of up-scaling these products from lab-based to an industrial scale (Figure 3). This section will primarily discuss factors and ways to improve the industrial relevance of FWBP in the packaging industry.

One advantage of active packaging is that it requires less amount of active compounds to be added to food due to its linear release over time [88]. The mechanism of active packaging is based on controlled release, in which the active packaging should release active compounds periodically until equilibrium is achieved between the packaging and the food over the storage time [95]. However, it is critical to ensure that the active packaging remains functional, at least throughout the storage of product. The storage stability of the film itself should be tested, and the release kinetics of the active compounds from the film matrix should be determined to ensure that shelf-life is prolonged and the lipid oxidation of food is inhibited.

Most research has focused on the development and the characterization of films or coatings. However, the application of these films or coating to the food should be tested to determine their effectiveness and performance. Chollakup et al. [56] showed an opposite antibacterial result when performed in vitro and on real food due to factors, such as the microbial mixture and load, the permeability of the packaging, the transfer rate of oxygen and water, and the food matrix. Therefore, the real efficiency on food products needs to be clarified before these films can be broadly adopted.

Biopolymers from FWBP are a popular alternative raw material to plastic packaging due to their biodegradability. Some biopolymers have been reported to degrade in several weeks to several months, depending on the temperature, moisture, and type of material [46]. Although the biodegradability of biopolymers is important for the environment, it should also be noted that the industry requires a material that is durable enough to ensure the main objectives of packaging are met, which are protection and containment of the packaged food. Therefore, to use biopolymers in the industry, their biodegradability needs to be thoroughly assessed in order that they can be used according to the estimated shelf-life of the product. For example, a highly biodegradable material can be used for short shelf-life food, such as ready-to-eat food, while materials that take a longer time to biodegrade can be used for longer shelf-life food, such as crackers or cookies.

Costs are of paramount importance in up-scaling lab-based research to an industrial size. However, most research has failed to address this concern. Still, research by Spiridon et al. [65] evaluated potential costs. An industrial process was simulated, and the costs to produce 1 kg of biocomposite films were calculated while considering market prices and energy input. This assessment assists in validating the relevance and potential of the product to be commercialized.

The concept of clean-label products using natural ingredients has gained the attention of consumers in recent years [96]. However, one of the main problems in clean-label products is extending their shelf-life and ensuring the safety of food without the use of synthetic ingredients, such as preservatives and decreasing the number of chemicals used [97]. The incorporation of FWBP in packaging could be a solution in developing clean-label products for several reasons: (1) It does not extend the list of additives used in the food product, since the active ingredients are in the packaging material and not in the food; (2) FWBP is a natural ingredient; thus, it conforms to the clean-label requirement; and (3) it improves the functionality of packaging in terms of preserving and extending the safety of food products in addition to the basic functions of packaging (protection, containment, and communication).

## 8. Conclusions

FWBP can have a huge potential in the food packaging industry as active packaging of natural origins, clean-label product, and for shelf-life extension of food. Many beneficial materials from FWBP can be valorized as active compounds, biopolymer materials, and pH indicators for the packaging industry. The utilization of FWBP in developing active (antioxidant and antimicrobial packaging) and intelligent packaging (colorimetric pH-sensitive film) could be an alternative to synthetic active substances, such as butylated hydroxyanisole, butylated hydroxytoluene, and artificial pH indicator. Antioxidant packaging incorporated with active compounds from FWBP, such as polyphenols can decrease the lipid peroxidation process by scavenging free radicals, which assists in prolonging the shelf life of food and reducing food spoilage. Antimicrobial packaging is another type of active packaging developed to inhibit or delay microbial spoilage in food. pH-sensitive films incorporated with natural pH indicator, such as anthocyanin showed an excellent colorimetric change that could indicate food spoilage and can be perceived easily by consumers. The applications of active and intelligent packaging on meat and seafood products justified the efficiency of these packaging in delaying the lipid oxidation process, slowing microbial growth, maintaining the quality of food throughout storage time, prolonging the shelf life of food, and detecting the spoilage of food. Furthermore, the usage of active packaging and intelligent packaging derived from FWBP did not impart negative sensorial properties and are widely accepted by consumers. However, there are several factors, such as storage stability of active and intelligent packaging, practicability, and cost evaluation, which need further assessment to make it more adaptable to the industry.

## Figures and Tables

**Table 1:** Percentage of discarded parts in food processing.

Source	Discarded Parts	Percentage of Discarded Parts	References
Potato	Peels	5–40%	[7]
Mango	Kernel and peels	35–55%	[8,9]
Tomato	Pomace	5–30%	[10,11]
Blueberry	Pomace	20–30%	[12,13]
Apple	Pomace	20–40%	[14]
Carrot	Pomace	30–50%	[15]

**Table 2:** Utilization of food waste and by-products as antioxidants and antimicrobial packaging.

Type of Activity	FWBP	Fabrication	Composition	Properties	References
Antioxidant	Olive leaf and pomace	Coating	2% chitosan 1% and 2% olive leaves extract 1% and 2% olive pomace extract	lower weight loss of coated-apple improved shelf-life stability	[28]
Antioxidant	Blueberry (BPE) and red grape skin pomace (GSP)	Film	2% chitosan 2% carboxymethyl cellulose 1%, 2%, and 4% BPE 1%, 2%, and 4% GSP	Film + GSP was more permeable than film + BPE Film + GSP has higher antioxidant activity than film + BPE	[24]
Antioxidant	Tomato pomace (TP)	Film	6% sodium caseinate 0 to 40% TP	Film + >20% TP increased 17–25% flexibility Film + TP reduced water absorption by >72%	[29]
Antioxidant	Banana peels (BP)	Coating	2% chitosan 4%, 8%, and 12% BP	Chitosan + BP has lower hydrophilicity Chitosan + BP has excellent antioxidant activity Coating improved the postharvest quality of apple	[30]
Antioxidant	Lime peels (LP)	Film	1% lime peel pectin Coconut water (CW) and glycerol 20% LP	Film + CW + LP has better water barrier properties LP increased the antioxidant activity of film	[31]
Antioxidant	Kiwifruit peels (KPE)	Film	3% watermelon rind pectin (WRP) 0.5%, 1%, and 1.5% KPE	WRP + KPE increased opacity, elongation at break, and water vapor permeability Chicken thigh wrapped in WRP + KPE film has lower lipid oxidation	[32]
Antioxidant	Mango peels (MPE)	Bilayer films	4% fish gelatin 5% MPE	Bilayer film (coating thickness of 60 μm) has better UV barrier and scavenging activity Bilayer film improved the oxidation stability of margarine for up to 28 days	[33]
Antioxidant	Litchi peels (LPE)	Coating	2% chitosan 0.3% nano TiO_2_ 3% LPE	Chitosan + nano TiO_2_ + LPE enhanced the water vapor barrier capacity, mechanical strength, and thermal stability LPE increased the total phenolic content and antioxidant capacity of film	[34]
Antioxidant	Coconut shells (CS)	Film	4:1 ratio of polyvinyl alcohol (PVA) to corn starch (ST) 3%, 5%, 10%, and 20% CSE Sepiolite clay (SP)	CS increased antioxidant activity of film up to 80% PVA + ST + CS + SP increased the thermal properties of films	[35]
Antioxidant	Grape peels (GPE)	Film	1 g konjac glucomannan (KGM) 10 mg of GPE 0%, 5%, 10%, and 15% carboxylation cellulose nanocrystal (CCNC)	KGM + CCNC + GPE improved water vapor barrier property and transparency KGM + 10% CCNC + GPE has the highest tensile strength	[36]
Antioxidant	Green apple skins (GAS)	Film	1.5 g methylcellulose (MC) 10%, 20%, and 25% freeze-dried GAS 10%, 20%, and 25% aqueous GAS	MC + GAS increased total phenolic content and antioxidant properties MC + aqueous GAS decreased tensile strength and increased elongation at break	[37]
Antioxidant	Mango peels (MP)	Film	4% fish gelatin 1%, 3%, and 5% MP	Film + MP has lower water vapor permeability and solubility Film + MP increased free radical scavenging activity	[38]
Antioxidant	Pineapple peels (PP)	Film	4 g of PVA 1 g of ST 5%, 10%, 15%, and 20% (PP)	Film + PP increased film thickness and water solubility Film + PP increased antioxidant activity	[39]
Antioxidant	Mango kernels	Film	0 to 20% mango kernel fat (MKF) 0 to 20% mango kernel phenolic extract (MKPE) 80 to 100% mango kernel starch	MKPE is better than MKF in decreasing water vapor permeability MKPE contributes active functions (antioxidant and UV absorbing) properties on films	[40]
Antioxidant	Corn silk (CSE) and black soybean seeds coat (BCCSE)	Film	1% shrimp shell waste protein 1% chitosan 1%, 3%, and 5% oolong tea (OTE) 1%, 3%, and 5% CSE 1%, 3%, and 5% BCCSE	Film + BSSCE increased UV barrier and has transparency value of 15.97 mm^−1^ OTE, CSE, and BCCSE increased antioxidant activity of film	[27]
Antioxidant	Rice straw	Film	40 g starch 2, 3, and 4 g antioxidant rice straw extract (RSE)	Film + RSE improved the oxygen barrier properties Film becomes more brittle with the increasing amount of RSE	[41]
Antimicrobial	Pomegranate peels (POP)	Coating	1% chitosan 0.5% locust bean gum 0.072, 0.180, and 0.361 g POP	Chitosan + POP reduced the *Pseudomonas* spp. count by about 2 log units and maintained the psychrotrophic microbial load below 7 CFU/g for 6 days	[42]
Antimicrobial	Orange peels (OP)	Film	1 g of WPI 2% gelatin 5% Cloisite 30B 7%, 14%, and 21% OP	Film + OP has higher antibacterial activity, tensile strength, and water solubility Film + 21% OP has the best antibacterial, mechanical, and physical properties	[43]
Antimicrobial	Red grape seeds (RGS)	Film	2 g of chitosan powder 3 g of gelatin powder 1% and 2% *Ziziphora clinopodioides* essential oil (ZEO) 1% and 2% RGS	Film + 2% ZEO + 2% RGS showed lowest bacterial growth, peroxide value, and total volatile base nitrogen content in fish samples Film + ZEO + RGS enhanced shelf life of fillet	[44]
Antimicrobial	Grapefruit seeds (GFS)	Film	poly(ε-caprolactone) (PCL) chitosan 0.5, 1.0, 1.5, 2.0, and 2.5 mL GFS	PCL + chitosan + GFS were effective against *Escherichia coli* and *Pseudomonas aeruginosa* PCL + chitosan + GFS inhibit the bacterial growth for up to 120 h	[45]

**Table 3:** Utilization of food waste and by-products as pH-sensitive film.

Source	Food Product	Color Changes	References
Red apple pomace	Salmon	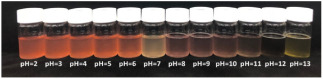 Color variation in pH buffer solution. 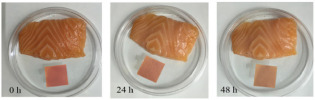 Color changes of film during storage of salmon.	[73]
Raspberry pomace	Pork skin	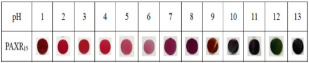 Color variation in pH buffer solution. 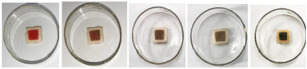 Color changes of film during storage of pork skin at 0, 6, 12, 24, and 48 h.	[74]
Grape skins	Pork	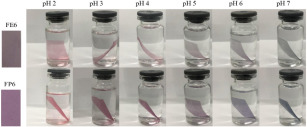 Color variation in pH buffer solution. 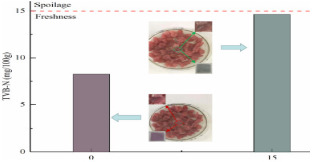 Color changes of film during storage of pork corresponding to TVBN value.	[68]
Black rice bran	Shrimp and hairtail	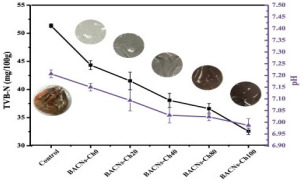 Color changes of film during storage of shrimp corresponding to TVBN value. 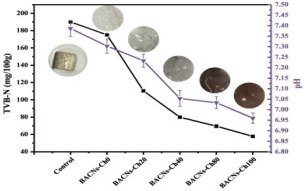 Color changes of film during storage of hairtail corresponding to TVBN value.	[20]
Black rice bran	Shrimp and pomfret meat	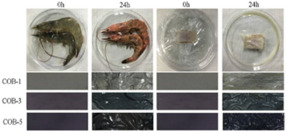 Color changes of film during storage of shrimp and pomfret meat.	[75]
Blueberry residue	Chicken meat	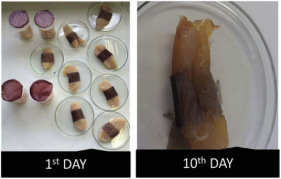 Color changes of film during storage of chicken.	[76]
*Vitis amurensis* husk	Fish	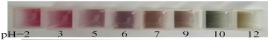 Color variation in pH buffer solution. 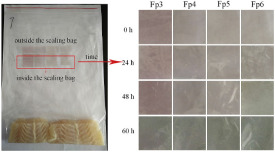 Color changes of film during storage of fish.	[77]

## Data Availability

Not applicable.
